# Unclassified mesenchymal sarcoma with *NTRK1-KHDRBS1* gene fusion: a case report of long-term tumor-free survival with crizotinib treatment

**DOI:** 10.1186/s12957-021-02237-y

**Published:** 2021-04-30

**Authors:** Weijie Chen, Huimei Wang, Dongxian Jiang, Lijuan Luan, Yuhong Zhou, Yingyong Hou

**Affiliations:** 1 Department of Pathology, Zhongshan Hospital, Fudan University, 180 Fenglin Road, Xuhui District, Shanghai, 200032 China; 2 Department of Medical Oncology, Zhongshan Hospital, Fudan University, Shanghai, 200032 China

**Keywords:** Mesenchymal sarcoma, Unclassified tumors, *NTRK1* gene fusion, Crizotinib, Treatment

## Abstract

**Background:**

Mesenchymal sarcomas are tumors that originate from mesenchymal tissue. Most mesenchymal sarcomas can be accurately classified, but some are unclassifiable in clinical practice. Molecular detection methods enable patients to benefit from molecular-targeted therapies for many cancers, including lung, breast, and bowel cancers. Further, even unclassified tumors can have therapeutic targets. NTRK gene fusions are sporadic genetic alterations that occur across tumor entities. If NTRK gene fusions are detected, TRK inhibitors can be used regardless of the tumor entity.

**Case presentation:**

This report describes a case with an unclassifiable mesenchymal sarcoma carrying a neurotrophic tyrosine receptor kinase *NTRK1-KHDRBS1* gene fusion that was diagnosed and treated at multiple hospitals. Diagnostic work-up included pathological and immunohistochemical analysis, which excluded angiosarcoma, dendritic cell sarcoma, and pseudomyogenic hemangioendothelioma. The patient achieved a long-term survival without tumor relapse after treatment with crizotinib.

**Conclusions:**

This case will be of significant interest to pathologists because, despite the tumor being unclassified, a molecular target was identified. Although the FDA does not currently approve crizotinib for treatment of patients harboring *NTRK* gene fusions, this case provides new insights for diagnosis and treatment of mesenchymal sarcomas with NTRK1 gene translocations. Similar to ALKomas, which can be successfully treated using NTRK molecular-targeted therapy, tumors with NTRK gene translocations can be classified as NTRKomas, even when they occur at different organ sites, and with varying histological morphologies, and immunophenotypes.

## Background

The neurotrophic tyrosine receptor kinase (NTRK) genes, *NTRK1*, *NTRK2*, and *NTRK3*, encode the tropomyosin receptor kinase (TRK) family of neurotrophin tyrosine kinase receptors, TRKA, TRKB, and TRKC, respectively [1], which have roles in development and normal functioning of the nervous system [2]. TRK proteins are activated through various biological mechanisms and are involved in malignant tumor development. Further, NTRK fusion genes can act as oncogenic drivers in cancer [3]. Many partner genes can fuse with NTRK, and NTRK fusions have been reported in several solid tumors in children and adults. A *TPM3-NTRK1* gene translocation was recently identified in colorectal cancer [4], and *TPM3-NTRK1* gene fusions have also been detected in rare tumors, such as breast secretory carcinomas and infantile fibrosarcomas. The U.S. Food and Drug Administration (FDA) has approved entrectinib and larotrectinib to treat patients with NTRK gene fusions [5].

Here, we report a case of a patient who presented with a mesenchymal malignant tumor on the top left side of his head. Results suggested that the tumor carried a fusion of exons 1–8 of the KHDRBS1 gene and exons 12–17 of the NTRK1 gene. The tumor regressed completely after the patient took crizotinib for 1 month. The patient is now in full remission, without disease progression, demonstrating that targeted can benefit patients following screening for molecular lesions. To our knowledge, no studies have yet reported any drugs that can treat tumors carrying an *NTRK1-KHDRBS1* fusion. This case report provides a reference for treating tumors with this gene fusion. Tumors carrying NTRK gene translocations can be classified as NTRKomas despite differences in organ site, histology, and immunophenotype.

## Case report

**Fig. 1:**
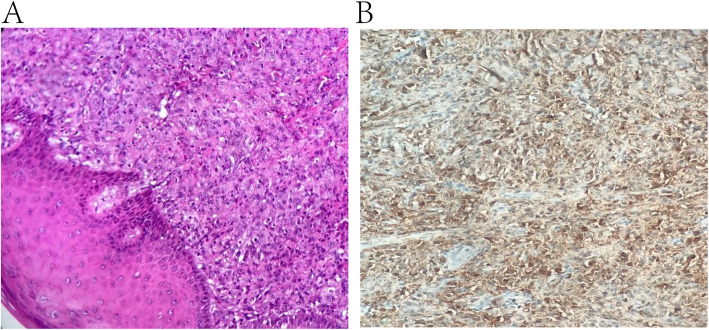
Microscopic appearance of a tumor sample showing abundant cell, with no tumor cells invading the epidermal layer of the skin (hematoxylin and eosin stained; ×20 magnification) (**a**). The immunohistochemical staining of tumor cells showing strong expression of S-100 (hematoxylin and eosin stained; ×20 magnification) (**b**)

A 33-year-old man presented to his primary care provider at Shanghai Shuguang Hospital with a left scalp mass. Biopsy showed a 2.5 × 1.5 × 1.0 cm mass with solid grayish-red coloring. Following a consultation at Zhongshan Hospital on November 15, 2016, a malignant skin tumor, with rich dense cells (Fig. 1a), moderately severe atypia, and a mitotic phase of approximately 50–60 mitotic cells/50 high-power fields, was identified, but was difficult to classify. Immunohistochemical staining showed strong s-100 expression in the tumor cells (Fig. 1b). Notably, the Ki-67 cell proliferation index was 80%; however, no expression of CD34, LCA, F8, EMA, SMA, ERG, MUM-1, CD21, CD35, or SOX-10 was detected.

**Fig. 2:**
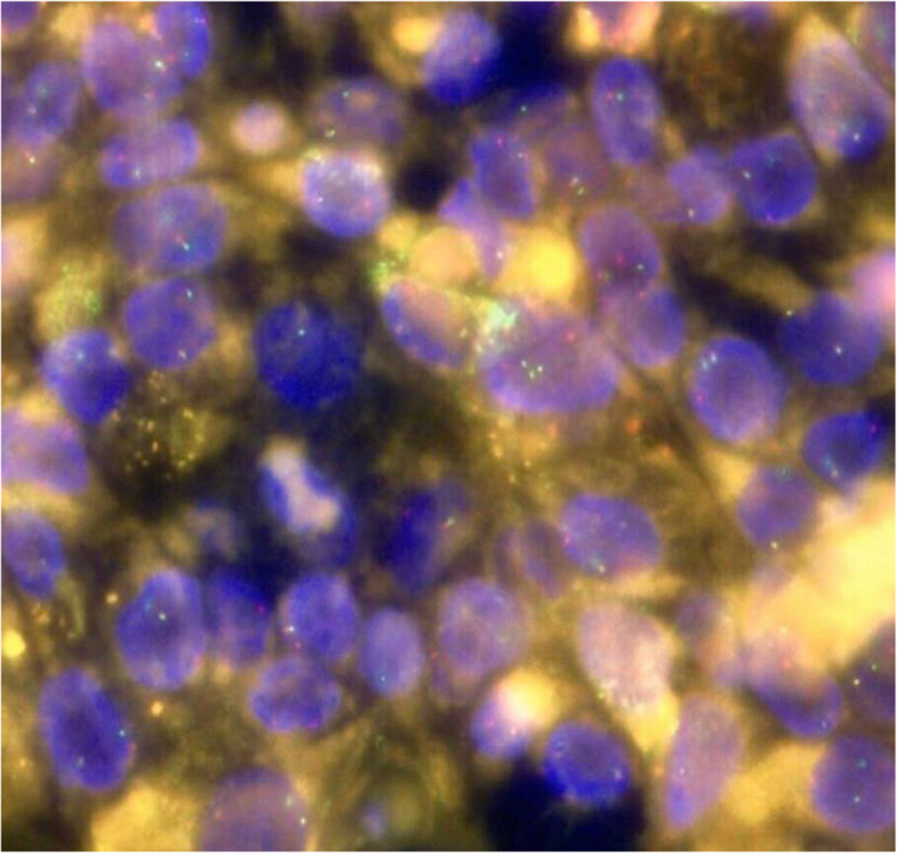
Fluorescence in situ hybridization showing the NTRK gene translocation

**Fig. 3:**
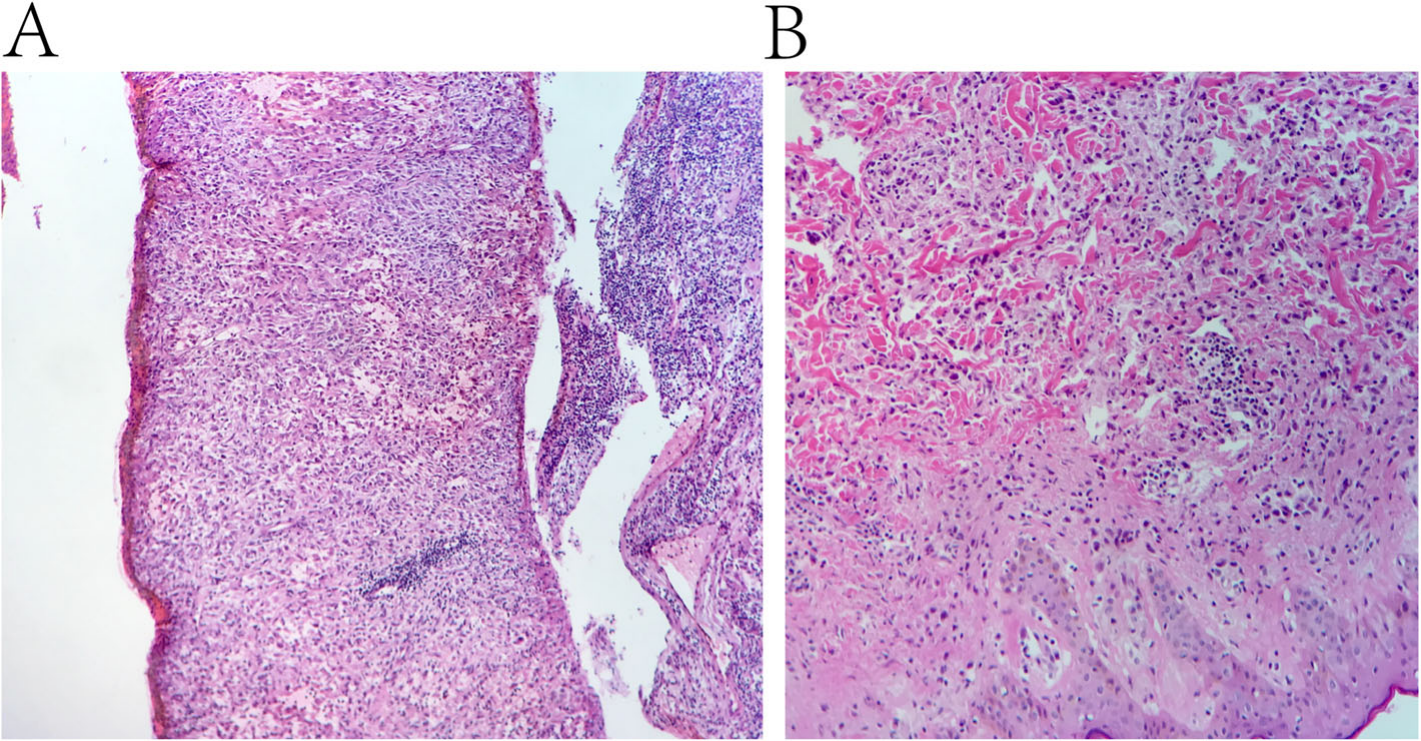
Biopsy results showing diffused dense tumor cells, with obvious medium-to-severe atypia (hematoxylin and eosin stained; ×4 magnification). Biopsy results showing round or oval tumor cells with unclear boundaries, obvious nucleoli, and active mitosis (hematoxylin and eosin stained; ×20 magnification)

**Fig. 4:**
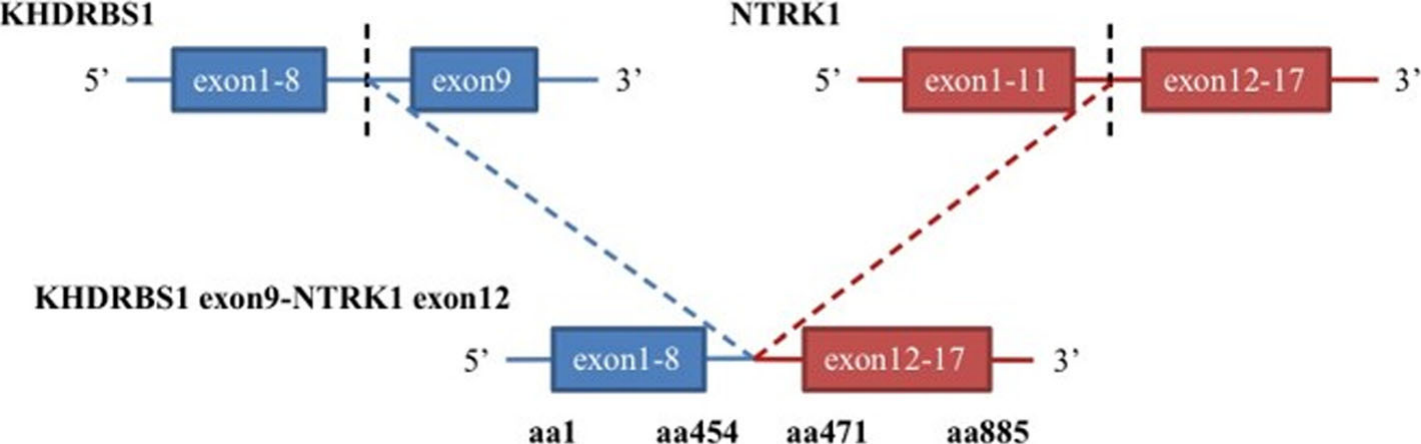
Next-generation sequencing (NGS) results showing that exons 1–8 of the KHDRBS1 gene and exons 12–17 of the NTRK1 gene were fused

Genetic testing revealed no fusions of the ALK, ROS-1, or RET genes. Furthermore, no mutations were detected in exons 18, 19, 20, or 21 of *EGFR*, exon 2 of *K-RAS*s, exons 2 or 3 of *N-RAS*, exon 15 of *B-RA*F, exons 9 or 20 of *PIK3CA*, or exon 20 of *HER*-2. The patient sought treatment at the National Cancer Center in Japan and was recommended for chemotherapy, but no standard regimen was available in January 2017. The patient then went to the Memorial Sloan-Kettering Cancer Center in the USA and was diagnosed with an unclassifiable mesenchymal sarcoma with NTRK1 gene translocation detected by next generation sequencing (NGS) (Fig. 2). In February 2017, the patient returned to Zhongshan Hospital with a left ear rash and a mass on the neck and upper right back. Biopsy revealed tumor metastasis and skin involvement. Microscopic examination revealed diffuse tumor cells, which were dense with clear moderate-to-severe atypia, round or oval with unclear boundaries, clear nucleoli, and active mitosis. Needle biopsy of tissue from tumor on the ear, neck, and back were consistent with that from the scalp tumor on the top left side (Fig. 3). NGS analysis indicated that exons 1–8 of the KHDRBS1 gene and exons 12–17 of the NTRK1 gene were genetically fused (Fig. 4).

**Fig. 5:**
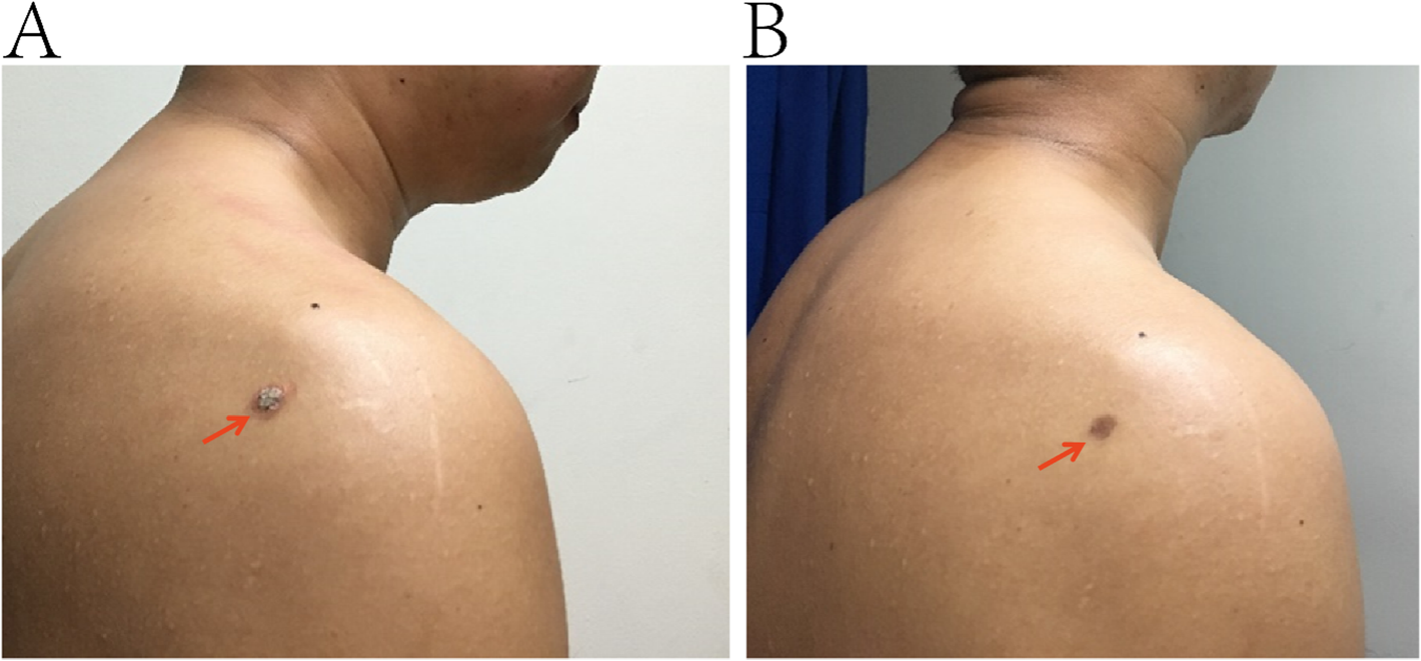
Rash on the right side of the patient’s back, which subsided after 1 month of treatment with crizotinib

Following a consultation with an oncologist and a pathologist in Zhongshan Hospital, Shanghai, China, the patient started on crizotinib (Pfizer; 250 mg/twice a day, orally) in February 2017 and was regularly followed-up at 3-month intervals to monitor his disease status. Moderate transaminase elevation, anorexia nausea, and vomiting occurred after 1 month of crizotinib; therefore, the drug dosage was reduced to 125 mg/twice a day for 2 months. The dosage was then restored to 250 mg/twice per day and reduced glutathione tablets taken daily to protect hepatic function. After 1 month, the lesion on the patient’s upper right back had subsided and flattened (Fig. 5). After 3 months, the left ear rash and neck mass completely disappeared. The patient was followed-up after taking crizonib for 3 months by observation of the skin surface lesions. He underwent general check-up every 6 months by imaging examination, fine needle aspiration, or biopsy, to determine whether there was disease recurrence. The most recent follow-up after taking crizotinib for 3 months by was in July 2020. The patient continued to show a complete response to crizotinib, without disease progression, according to examination of visually apparent superficial lesions, pathological assessment, and radiography. The patient is currently taking crizotinib; however, it is not planned for this treatment to continue indefinitely. The long-term sequelae of crizotinib use are likely respiratory system damage, gastrointestinal system damage, heart rate and rhythm disorders, and inevitable acquired drug resistance. Crizotinib will be stopped under medical’s supervision once acquired drug resistance or severe adverse reactions occur.

## Discussion

Soft tissue tumors originate in nonepithelial tissues, which include bone, lymphatic, hematopoietic, glial, fibrous, adipose, striated muscle, vascular, and peripheral nervous tissues. Immunohistochemical techniques, NGS, and fluorescence in situ hybridization have revealed the molecular profiles of many soft tissue tumors [6]. Soft tissue sarcomas are malignant tumors derived from mesenchymal tissue; such sarcomas frequently relapse and are difficult to treat after surgery. Pathological reports of these tumors are relatively rare, particularly of cases with NTRK1-KHDRBS1 gene fusion.

NTRK gene translocations are rarely encountered in tumors in routine clinical practice. NTRK genes encode the TRK family of receptor tyrosine kinases [7], with *NTRK1*, *NTRK2*, and *NTRK3* encoding TRKA, TRKB, and TRKC, respectively. The TRK receptors homodimerize and activate multiple signal transduction pathways only when stimulated by their ligands (nerve growth factor, brain-derived growth factor, and NT-4/5) and influence target genes involved in cell survival, differentiation, and proliferation [8]. The TRKA receptor has a high affinity for nerve growth factor and is involved in production and maintenance of pain in humans [9]. NTRK gene fusions caused by chromosomal mutations can lead to abnormal regulation of signaling pathways downstream of TRK receptors. Fusion between the NTRK tyrosine kinase domain and its partners via genetic rearrangement generates a chimeric TRK protein without the ligand-binding domain, which leads to overexpression by constitutive activation, or an always-on state, of the TRK kinase domain. This generates increased downstream signaling, cell proliferation, and cell growth. NTRK fusions typically occur at low frequencies (commonly<1%) in various common tumors and at high frequencies (≥90%) in rare cancers, such as secretory carcinomas of the breast or salivary gland and infantile fibrosarcoma [10, 11].

Oncogenic drivers have been detected in multiple cancers, allowing the development of targeted therapeutic approaches. NTRK gene fusions are oncogenic drivers, which can be targeted with TRK inhibitors. Further, clinical trials in large cohorts showed that larotrectinib (formerly known as LOXO-101) exhibited marked and durable antitumor activity in both adult and pediatric patients with TRK fusion positive cancer and locally advanced or metastatic solid tumors, regardless of patient age or tumor type, although resistance inevitably developed. Subsequently, then larotrectinib was approved on November 27, 2018, by the FDA as a medicine showing substantial clinical benefits in both pediatric and adult patients harboring NTRK gene fusions [12]. Further, larotrectinib is the first and most-studied selective pan-tyrosine kinase inhibitor (TKI) and is highly selective against TRKA, TRKB, and TRKC fusion proteins [13]. Meanwhile, it was reported at ESMO 2018 that entrectinib, a small molecule that inhibits TRK, ROS1, and ALK rearrangements, can induce clinically meaningful and durable responses in NTRK-fusion-positive solid tumors, regardless of type and whether or not disease has spread to the central nervous system [14].

Crizotinib, an oral, ATP-competitive TKI-effective drug, is another TKI-targeting agent currently under investigation. The FDA authorized crizotinib to treat anaplastic lymphoma kinase (ALK)-rearranged non-small cell lung cancer (NSCLC) in August 2011, only 4 years after the first published report of an ALK-rearranged NSCLC [15]. Crizotinib is highly effective against ROS-1-rearranged lung cancer and has been used to treat this cancer clinically [16]. A case reported in 2019 revealed a durable clinical response to crizotinib in an NSCLC carrying an *IRF2BP2-NTRK1* fusion [17]. Literature reports indicate that the most common adverse drug reactions involving organ systems following crizotinib treatment were damage to the liver, digestive, urinary, and blood and circulatory systems. Serious adverse reactions to crizotinib have also included elevated transaminase, sinus bradycardia and myelosuppression. Other adverse reactions, which may recover spontaneously include nausea, vomiting, loss of appetite, and blurred vision [18]. Compared with larotrectinib or entrectinib, crizotinib is a less potent inhibitor, despite strongly inhibiting TRKs at low nanomolar concentrations in vitro, which may explain why the FDA has not approved crizotinib for treatment of patients with tumors harboring NTRK gene fusions. Nevertheless, there are case reports of crizotinib use for treatment of tumors with NTRK gene fusions [19]. The patient in the present case reported that he could not afford larotrectinib; therefore, he was treated with crizotinib. One month later, his skin metastases had flattened and decreased significantly; however, he had also developed moderate transaminase elevation, anorexia, nausea, and vomiting. The drug dosage was reduced and liver protective drugs administered and liver function returned to normal after 2 months. He has not relapsed after 45 months, demonstrating that the tumor cells showed good, persistent sensitivity to crizotinib. No related reports have previously been published describing targeted drug treatment of tumors with *NTRK1-KHDRBS1* gene fusions. The patient reported here continues to receive therapy. In summary, after only 1 month of treatment, his illness resolved and has not recurred in approximately 4 years.

## Conclusion

In conclusion, this paper reports a rare case with a mesenchymal sarcoma carrying an *NTRK1* gene translocation. The patient reported that he could not afford larotrectinib; therefore, he was treated with crizotinib monotherapy, which effectively controlled disease progression. Compared with larotrectinib or entrectinib, crizotinib inhibits TRKs less potently at low nanomolar concentrations in vitro, which may be why the FDA has not approved crizotinib for treatment of tumors harboring NTRK gene fusions. This case provides new insights potentially helpful for diagnosing and treating mesenchymal sarcomas with *NTRK1* gene translocation. Further, these findings indicate that molecular targets should be screened in cases with unclassifiable tumors.

## Data Availability

Not applicable

## Abbreviations

NTRK: Neurotrophic tyrosine receptor kinase
TRK: Tropomyosin receptor kinase
FDA: Food and Drug Administration
NGS: Next-generation sequencing
TKI: Pan-tyrosine kinase inhibitor
ALK: Anaplastic lymphoma kinase
NSCLC: Non-small cell lung cancer
